# Characteristics of Quinoa Protein Isolate Treated by Pulsed Electric Field

**DOI:** 10.3390/foods13010148

**Published:** 2024-01-01

**Authors:** Xinyue Zhang, Zhanrong Li, Xiaojiao Zheng, Wenjun Wen, Xiaowen Wang

**Affiliations:** 1 Food Science and Engineering College, Shanxi Agricultural University, 1 Mingxian South Road, Taigu District, Jinzhong 030801, China; echogo2023@163.com (X.Z.); lzr19980817@163.com (Z.L.); z13133128694@163.com (X.Z.); wenwenjun@sxau.edu.cn (W.W.); 2 Houji Laboratory in Shanxi Province, No. 81 Longcheng Street, Xiaodian District, Taiyuan 030031, China

**Keywords:** pulsed electric field treatment, quinoa protein isolate, structural and functional properties

## Abstract

The aim of this study was to investigate the impact of a pulsed electric field (PEF) on the structural and functional properties of quinoa protein isolate (QPI). The findings revealed a significant alteration in the secondary structure of QPI following PEF treatment, converting the random coil into the β-sheet, resulting in an improvement in structure orderliness and an enhancement of thermal stability. The PEF treatment led to a reduction in particle size, induced structural unfolding, and increased the surface hydrophobicity, resulting in a statistically significant enhancement in the solubility, foaming, and emulsifying properties of QPI (*p* < 0.05). Specifically, PEF treatment at 7.5 kV/cm for 30 pulses was identified as the optimal condition for modifying QPI. This study provides a basis for the precision and range of application of pulsed electric field treatment and offers the possibility of improving the physical and chemical properties of quinoa protein.

## 1. Introduction

Quinoa (*Chenopodium quinoa* Willd.) has a long history of cultivation and consumption. Quinoa also has a high tolerance to climatic extremes such as frost and drought and soil conditions such as high salinity [1], which gives it great application prospects in agricultural cultivation and food processing [2]. With the development of modern society and the pursuit of a healthy diet by consumers, the global trend of quinoa consumption has gradually expanded [3]. Quinoa has a protein content ranging from 15.6% to 18.7% [4]. The biological value of quinoa protein is 73%, comparable to that of beef (74%) and much higher than that of other crops such as rice (56%) [5]. Quinoa is considered a protein-rich food and rich in lysine (59 g/kg), which is lacking in most cereals [6]. Quinoa is a gluten-free food and is a suitable food for people suffering from celiac disease [7]. Compared with animal albumin, plant proteins perform poorly in functional properties, such as solubility [8]. More research is needed to improve the processing characteristics of quinoa protein to achieve its commercial exploitation and utilization.

In recent years, nonthermal treatments (ultrasonic, high-pressure microfluidics, and pulsed electric field treatments) have attracted widespread attention because of their low energy consumption, environmental friendliness, and maximized retention of food nutrients [9]. Nonthermal treatments can assist or substitute for traditional food processes and provide consumers with a healthier and higher-quality experience [10]. As a burgeoning and potential nonthermal food processing technology, PEF is progressing from the laboratory to industrial production [11]. Studies on the application of PEF in food processing have shown that it can inactivate microorganisms [12] and extract active ingredients [13], modify biomacromolecules [14], enhance chemical reactions [15], and accelerate the ripening of fermented foods [16]. In the processing of proteins, PEF treatment can induce the cooperative movement of proteins on the temporal scale of microseconds, and molecular rearrangement of proteins by the alternating force produced by high-frequency pulse polarity reversal [17], which can change the structure of proteins and then change their properties [18]. Studies have indicated that a PEF treatment (>30 kV/cm, 288 μs) can modify soybean protein isolate by inducing dissociation, denaturation, and repolymerization of the protein, leading to changes in characteristics by altering the spatial structure of the protein [19]. PEF treatment has the potential to become a key technology for protein food processing in the future [20]. However, more research data and functional optimization are needed to realize the application potential of PEF in food industrial production.

In this study, we investigated and clarified the mechanism of a pulsed electric field on the characteristics of QPI, and provided a basis for the application precision and range of pulsed electric field treatment. In addition, this study has provided a new idea for improving the structural and functional properties of quinoa protein. 

## 2. Materials and Methods

### 2.1. Materials

Quinoa seed was obtained from Shanxi JiaQI Agricultural Technology Co., Ltd. (Shanxi, China). N-hexane was obtained from Aladdin Biochemical Technology Co., Ltd. (Shanghai, China). Sodium dodecyl sulfate (SDS), Coomassie Brilliant Blue G-250 destaining solution, β-mercaptoethanol (BME), protein marker (14.4–97.4 kD), and 1-anilino-8-naphthalene sulfonate (ANS) were obtained from Beijing Solaibao Technology Co., Ltd. (Beijing, China). All other chemical reagents were of analytical grade. 

### 2.2. Extraction of a Native Quinoa Protein Isolate (N-QPI)

The method of extraction of the quinoa protein isolate refers to Alrosan et al. [21]. Quinoa was milled into flour and defatted twice with 1:5 (*w*/*v*) n-hexane. The degreased quinoa flour and deionized water mixture at 1:12 (*w*/*v*) was adjusted to pH 11 (NaOH, 0.1 mol/L) and stirred at 45 °C for 3 h. Then, the sample was centrifuged (4000 r/min, 20 min), and the supernatant was collected (adjusted pH to 4.5, HCl, 0.1 mol/L). After standing for 30 min, the supernatant was centrifuged for 20 min (4000 r/min), the sediment was collected, washed twice with deionized water, centrifuged (4000 r/min, 20 min), and the sediment pH was adjusted to 7.0 (NaOH, 0.1 mol/L). Then, the sample was lyophilized and crushed to obtain the native quinoa protein isolate (N-QPI). The protein content of N-QPI was measured as 83.2% using the Kjeldahl method (Method 930.29, N × 6.25) [22]. 

### 2.3. PEF Treatment of Quinoa Protein Isolate (PEF-QPIs)

The PEF processing system was set up by Xin ‘an Food Technology Co., Ltd. (Guangzhou, Guangdong, China) [23]. N-QPI was dissolved in deionized water (5% *w*/*w*). The suspension was treated by 10, 30, and 50 pulses at 7.5 kV/cm field strength, with an interval of 0.08 ms and a wavelength of 0.052. Then, the samples were lyophilized and crushed to obtain the PEF-QPIs (7.5 kV/cm 10, 30, 50 pulses) and stored at −20 °C until use. 

### 2.4. Scanning Electron Microscopy (SEM)

The microstructure images of N-QPI and PEF-QPIs were obtained by SEM (JSM-7500, JEOL, Tokyo, Japan) based on the method described by Zhang et al. [24]. The N-QPI and PEF-QPIs (7.5 kV/cm 10, 30, 50 pulses) obtained in Section 2.2 and Section 2.3 were uniformly stuck to the conductive tape separately and sputter-coated under a high vacuum with gold. The acceleration of voltage was 10 kV.

### 2.5. Determination of Particle Size and Zeta Potential

The particle size and zeta-potential measurements were based on the method described by Lyu et al. [25]. The N-QPI and PEF-QPIs (7.5 kV/cm 10, 30, 50 pulses) obtained in Section 2.2 and Section 2.3 were dissolved in deionized water (1 mg/mL). The zeta potential and particle size were determined after magnetic stirring at 25 °C for 2 h (Zetasizer Nano ZS90, Malvern, PA, USA).

### 2.6. SDS-PAGE Analysis

The SDS-PAGE procedure of operation was based on the method described by Mir et al. [26]. Acrylamide separation and concentration gel were 12% and 4% apart, respectively, and used for SDS-PAGE analysis. The N-QPI and PEF-QPIs (7.5 kV/cm 10, 30, 50 pulses) obtained in Section 2.2 and Section 2.3 were mixed with sample buffer and bromophenol blue reagent (5 mg/mL), respectively, stirred at 25 °C for 2 h, and boiled in a the water bath for 10 min. In addition, 20 μL/mL β-mercaptoethanol along with the sample buffer was used for reduction electrophoresis. The SDS-PAGE analysis used 14.4–97.4 kDa mixed protein markers (Beijing Solaibao Technology Co., Ltd., Beijing, China) as a reference. The operating voltage was 100 V for the sample concentrate and 150 V for the sample separate at a steady current of 40 mA (DYY-7C electrophoresis apparatus, Beijing, China). After electrophoresis, the gel was stained with Coomassie Brilliant blue G-250 for 2 h and decolorized with the destaining solution for 6 h and tap water for 2 h.

### 2.7. Determination of Fourier-Transform Infrared Spectroscopy

The Fourier-transform infrared spectroscopy measurement was based on the method described by Ling et al. [27]. The secondary structure of the samples was determined using FTIR spectrometer (Tesor 27, Bruker Co., Bremen, Germany). The N-QPI and PEF-QPIs (7.5 kV/cm 10, 30, 50 pulses) obtained in Section 2.2 and Section 2.3 were separately mixed with KBr (1:200, *m*/*m*) to analyze the spectra with a wavelength range of 4000–400 cm^−1^. Omnic and Peak Fit v4.12 software were used to analyze the spectra. 

### 2.8. Determination of UV Spectrum

The UV spectrum measurement was based on the method described by Liu et al. [28]. The N-QPI and PEF-QPIs (7.5 kV/cm 10, 30, 50 pulses) obtained in Section 2.2 and Section 2.3 were separately dissolved in deionized water (1 mg/mL) and stirred at 25 °C for 2 h, and the UV spectrum of 200–400 nm was measured by a UV–visible spectrophotometer (Agilent Technologies Co., Ltd., Shanghai, China). Deionized water was left as the blank. The scanning rate was 100 nm/min, the scanning interval time was 0.25 s, the scanning width was 1.0 nm, and the scanning minimum interval was 0.2 nm. Five scans were averaged to obtain the final UV spectrum data. 

### 2.9. Determination of Intrinsic Fluorescence Spectrum

The intrinsic fluorescence spectrum measurement was based on the method described by Liu et al. [28]. The N-QPI and PEF-QPIs (7.5 kV/cm 10, 30, 50 pulses) obtained in Section 2.2 and Section 2.3 were separately dissolved in deionized water (1 mg/mL) and stirred at 25 °C for 2 h. The emission wavelength was in the range of 300–400 nm, and the excitation wavelength was 290 nm, as measured by a Fluorospectro Spectrometer (RF-5301 Shimadzu International Trading Co., Ltd., Shanghai, China). Deionized water was left as the blank. 

### 2.10. Determination of Surface Hydrophobicity

The surface hydrophobicity measurement was based on the method described by Mir et al. [26]. The N-QPI and PEF-QPIs (7.5 kV/cm 10, 30, 50 pulses) obtained in Section 2.2 and Section 2.3 were separately dissolved in deionized water (0.5 mg/mL), and gradient dilution ranged from 0.05 mg/mL to 0.5 mg/mL. ANS (8 mmoL, 20 μL) was added to each above-diluted sample (4 mL), and the mixture was reacted in the dark for 10 min. The excitation wavelength was 390 nm, the emission wavelength was 470 nm, and the slit width was 5.0 nm, as measured by a Fluorospectro Spectrometer (RF-5301 Shimadzu International Trading Co., Ltd., China). The protein concentration was plotted against the fluorescence intensity, and the surface hydrophobicity index was the slope of the curve calculated by linear regression.

### 2.11. Differential Scanning Calorimetry

The differential scanning calorimetry (DSC) measurement was based on the method described by Zhang et al. [24]. The N-QPI and PEF-QPIs (7.5 kV/cm 10, 30, 50 pulses) obtained in Section 2.2 and Section 2.3 were separately added to a standard aluminum pot (3 mg), sealed and equilibrated at 25 °C for 4 h. At a rate of 10 °C/min, the samples heated in DSC were heated from 30 °C to 130 °C (Mettler-Toledo, Greifensee, Switzerland) under a nitrogen purge environment (50 mL/min). 

### 2.12. Determination of the Functional Properties of the Quinoa Protein Isolates

#### 2.12.1. Determination of Solubility 

The solubility measurement was based on the method described by Zhang et al. [29]. The N-QPI and PEF-QPIs (7.5 kV/cm 10, 30, 50 pulses) obtained in Section 2.2 and Section 2.3 were separately dissolved in deionized water (10 mg/mL). The mixture was magnetically stirred at 25 °C for 2 h, centrifuged (4000 r/min, 20 min), and the solubility of the supernatant was determined by the Kjeldahl nitrogen determination method. The absorbance was measured at a wavelength of 540 nm. P is the protein solubility, %; N_0_ is the total protein content, mg/mL; N_1_ is the protein content in the supernatant, mg/mL. The determination of solubility was calculated with the following equation:

(1) $$P(\%)=(N_{1}/N_{0})\times 100$$

#### 2.12.2. Determination of Emulsifying Properties 

The measurement of the emulsifying properties was based on the method described by Chen et al. [30]. The N-QPI and PEF-QPIs (7.5 kV/cm 10, 30, 50 pulses) obtained in Section 2.2 and Section 2.3 were separately dissolved in deionized water (10 mg/mL). The mixture was magnetically stirred at 25 °C for 2 h and homogenized with soybean oil (3:1 *v*:*v*) by FSH-2a adjustable high-speed homogenizer for 1 min (10,000 rmp/min, Hangzhou Research and Experimental Instrument Co., Ltd., Hangzhou, China). Then, 100 μL from the emulsion was sampled to 0.1% SDS solution (10 mL), and vortexed for 5 s. The absorbance was measured at the wavelength of 500 nm, and measured again after 30 min. A_0_ is the absorbance of the sample at 500 nm wavelength for 0 min; D is the dilution coefficient (40); N_0_ is the initial protein concentration (g/mL); φ is the optical path length (1 cm); θ is the fraction of oil used to form the emulsion (0.25); ΔA is the change of absorbance from 0 to 30 min; t is the time interval (30 min). The EAI (emulsion activity index) and ESI (emulsion stability index) were calculated with the following equation:

(2) $$EAI(m^{2}/g)=(2\times 2.303\times A_{0}\times D)/(N_{0}\times \phi \times \theta \times 10000)$$

(3) $$ESI(\min)=(A_{0}/\Delta A)\times t$$

#### 2.12.3. Determination of Foaming Properties 

The foaming capacity (FC) and foaming stability (FS) measurements were based on the method described by Lyu et al. [25]. The N-QPI and PEF-QPIs (7.5 kV/cm 10, 30, 50 pulses) obtained in Section 2.2 and Section 2.3 were separately dissolved in deionized water (10 mg/mL). The mixture was magnetically stirred at 25 °C for 2 h and homogenized by FSH-2a adjustable high-speed homogenizer for 2 min (10,000 rmp/min, Hangzhou Research and Experimental Instrument Co., Ltd.). The FC was measured by comparing foam volume after homogenizing with the sample liquid volume. The FS was measured after 30 min in the same way as the FC. V_0_ is the volume of foam after homogenization, mL; V is the initial volume of quinoa protein solution, mL; V_30_ is the foam volume measured after standing for 30 min, mL. The determination of FC and FS was calculated with the following equation:

(4) $$FC(\%)=(V_{0}/V)\times 100$$

(5) $$FS(\%)=(V_{30}/V_{0})\times 100$$

### 2.13. Statistical Analyses 

The indices involved in the experiment were measured three times. The mean value and standard deviation of the data were analyzed by SPSS 17.0. Graphs were drawn by Origin 8.5. The FTIR spectra were analyzed by Peak Fit V4.12.

## 3. Results and Discussion 

### 3.1. Effect of PEF Treatment on Scanning Electron Microscopy (SEM) of QPIs

**Figure 1:**
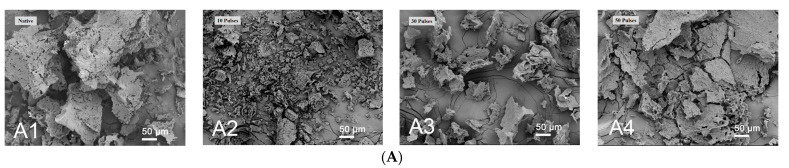

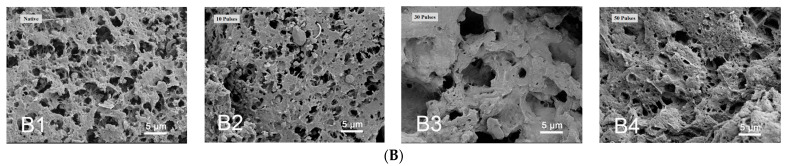
SEM images of QPIs. SEM images of QPIs at 50 μm plotting scale ((**A**): (**A1**) shows native quinoa protein isolate (N-QPI); (**A2**–**A4**), from left to right, treated with 10, 30, and 50 pulses of PEF at 7.5 kV/cm) and at 5 μm plotting scale ((**B**): (**B1**) shows N-QPI; (**B2**–**B4**), from left to right, treated with 10, 30, and 50 pulses of PEF at 7.5 kV/cm).

Figure 1A shows that N-QPI was in the form of large particles, and PEF treatment with 7.5 kV/cm 10 pulses caused quinoa protein to fragment into small-diameter particles, which may be due to the lower number of treatments (7.5 kV/cm 10 pulses group) shattering the aggregates of quinoa protein and making its particles smaller. The reduction of the particle size is conducive to the improvement of solubility [24]. However, PEF treatment with 7.5 kV/cm 30 and 50 pulses resulted in a formation of aggregates and smaller particles of quinoa protein, which was consistent with the particle size change of samples under this condition, and the solubility decreased with the increase of particle size. In Figure 1B, N-QPI is a porous honeycomb structure. The PEF treated QPI with 7.5 kV/cm 10 pulses decreased in aperture and inflated in volume. The display of images of PEF-QPI treated with approximately 30 and 50 pulses is less porous but fluffy and rough. The 7.5 kV/cm 30-pulse treatment had a positive effect on the compactness and uniformity of the QPI’s structure, leading to an ordered matrix with a smaller void, conducive to the formation of a stable interfacial layer at the gas–liquid interface and the adsorption capacity of proteins at the oil–water interface [28]. However, excessive processing (7.5 kV/cm 50 pulses) will destroy this stable structure and reduce the protein’s foaming and emulsifying properties [24]. The results show that the PEF treatment altered the QPI particle size and microstructure of quinoa proteins. The more pulses used in QPI treatment, the greater the structural changes of quinoa protein. Zhang et al. [29] reported the transformation of rapeseed protein after pulsed electric field treatment, and along with the increase in treatment intensity, rapeseed protein also produced significant aggregates, similar to the results of this experiment.

### 3.2. Effect of PEF Treatment on the Particle Size and Zeta Potential of QPIs

**Figure 2:**
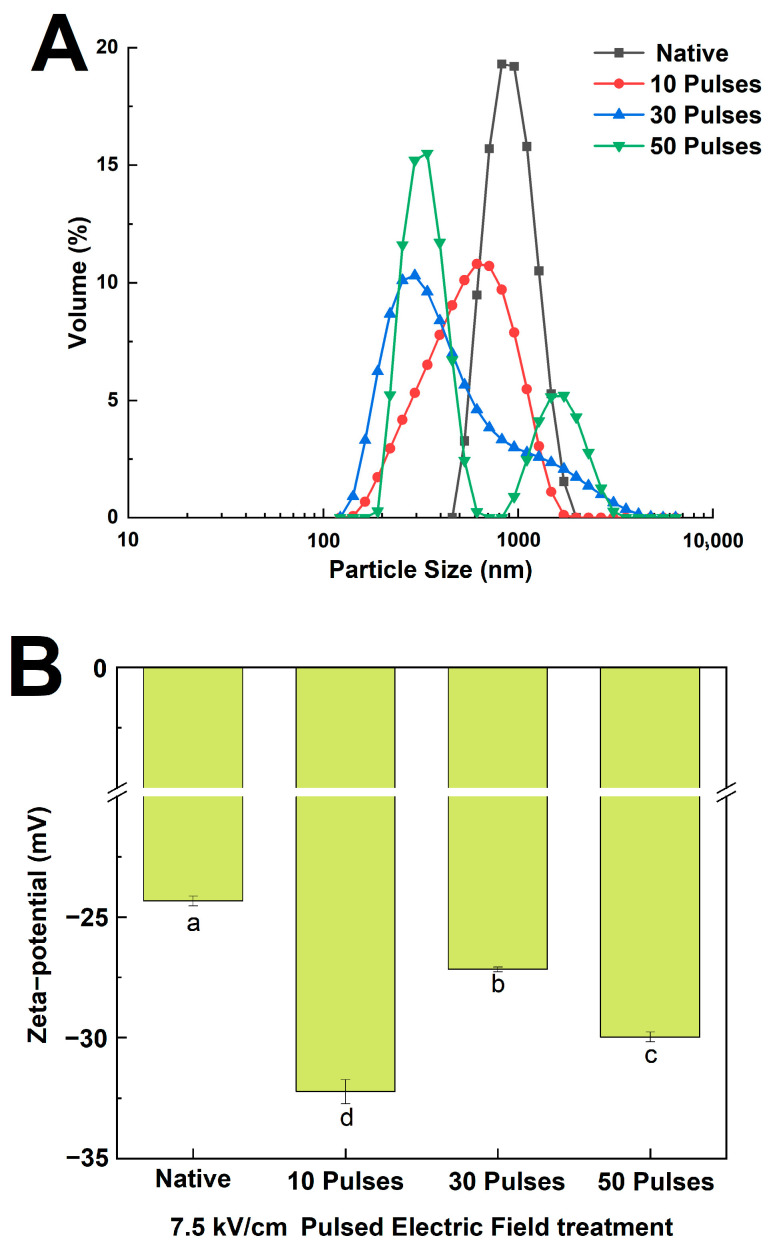
Particle size distribution (**A**) and zeta potential (**B**) of N-QPI and 10, 30, and 50 pulses under 7.5 kV/cm-treated PEF-QPIs. All experiments were repeated three times, and different letters indicate significant differences (*p* < 0.05).

The particle size distribution reflects the effect of pulsed electric field treatment on quinoa protein particle size. The results in Figure 2A show that PEF treatment causes quinoa proteins to migrate toward smaller particle sizes. The peak size distribution of N-QPI was 825 nm, 615 nm for PEF-QPI 7.5 kV/cm 10 pulses, and PEF-QPI 7.5 kV/cm 50 pulses had two peak size distributions of 340 nm and 1720 nm, respectively. The results above indicated that some quinoa proteins formed aggregates and smaller particles after treatment with 7.5 kV/cm 50 pulses, which changed the particle size distribution of quinoa proteins. The change above was similar to that observed by SEM.

The pH of the PEF-QPIs and N-QPI samples was 7.0, which is higher than the isoelectric point, so the zeta potential of the QPIs was negative. The results in Figure 2B show that the absolute value of zeta potential in 7.5 kV/cm 10, 30, and 50 pulses PEF-QPIs (32.23, 27.17, and 29.97 mV) was significantly higher than that in N-QPI (24.33 mV). Compared with N-QPI, PEF treatment at low pulses (7.5 kV/cm 10 pulses) reduced the particle size of QPI, exposed the negatively charged groups to the outside, strengthened the repulsive force of PEF-QPIs, and increased the absolute value of zeta potential. With the increase in treatment pulses (7.5 kV/cm 30, 50 pulses), QPIs formed partial aggregates, protein particles enlarged, and the absolute value of zeta potential declined. These results indicate a correlation between protein aggregation and zeta potential [30].

### 3.3. SDS-PAGE Analysis of N-QPIs and PEF-QPIs 

**Figure 3:**
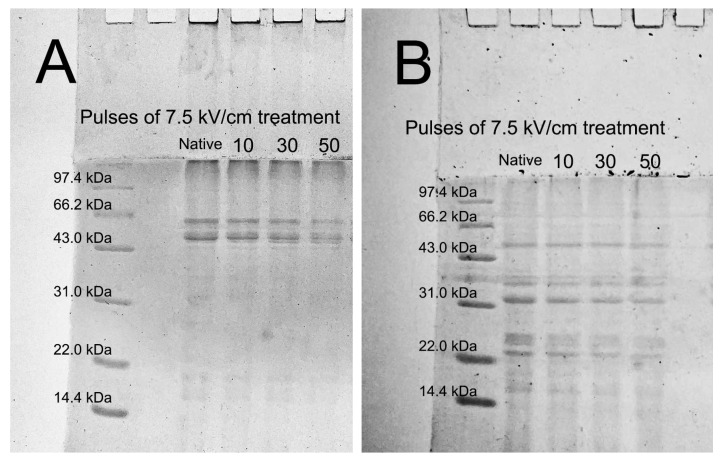
Electrophoresis patterns of unadded β-mercaptoethanol (**A**) and added β-mercaptoethanol (**B**) of N-QPI and 10, 30, and 50 pulses under 7.5 kV/cm-treated PEF-QPIs.

Chenopodium, an 11S-type globulin, is the main component protein of QPI [31]. Figure 3A shows that the quinoa protein before and after PEF treatment mainly had two distinct bands between 43.0 and 66.2 kDa, which is consistent with Makinen’s report that the quinoa protein is composed of 49 and 57 kDa subunits (AB-11S) that were associated with a hexamer by noncovalent interactions [32]. PEF treatment caused no changes in the molecular size of quinoa proteins, indicating that pulsed field treatment caused no change to the primary structure of quinoa proteins. The addition of β-mercaptoethanol can break the disulfide bond and result in subunit bands with low molecular weights. Figure 3B shows that β-mercaptoethanol reduced the quinoa protein from 49 to 57 kDa to several acidic subunits (AS) between 31.0 kDa and 43.0 kDa and to several basic subunits (BS) between 14.4 kDa and 31.0 kDa [33]. No significant band changes were observed before and after PEF treatment, indicating that the different pulses of PEF treatments did not alter the linkage sites of the quinoa protein disulfide bond. Ling et al. [27] also reported that radiofrequency treatment of rice bran protein isolate did not reduce the number of subunit bands or the intensity of the protein profile. 

### 3.4. Effect of PEF Treatment on the Secondary Structure of QPIs

**Figure 4:**
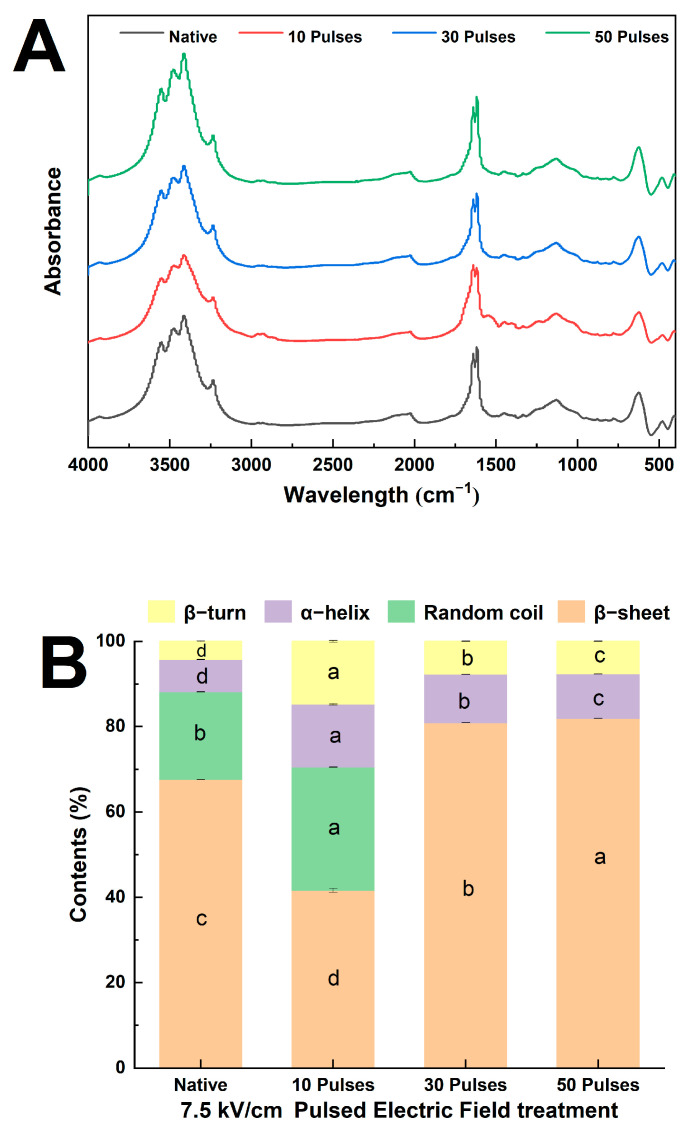
Fourier-transform infrared line spectrum (**A**) and secondary structure percentage stacking plot (**B**) of N-QPI and 10, 30, and 50 pulses under 7.5 kV/cm-treated PEF-QPIs, all experiments were repeated three times, and different letters indicate significant differences with same structure (*p* < 0.05).

Figure 4A shows the FTIR of PEF-QPIs and N-QPI recorded at 500–4000 cm^−1^, which had large numbers of characteristic absorption peaks between 3250 and 3750 cm^−1^. The above characteristic absorption peaks were generally considered to be stretching vibrations of hydrogen bonds in the protein skeleton (N-H, O-H) [26]. The FTIR spectra of the four samples showed no significant red or blueshifts and the characteristic broad absorption peaks were similar, indicating that the PEF treatment caused no alteration to the secondary structure skeleton of quinoa proteins.

Analysis of protein FTIR spectra using Gaussian curve matching combined with the second derivative of Fourier self-convolution and deconvolution images can reveal information about protein group and microenvironment changes and determine the intermolecular forces that may exist. The characteristic absorption peaks are generally considered to be 1600–1640 cm^−1^ for β-sheets, 1640–1650 cm^−1^ for random coils, 1650–1660 cm^−1^ for α-helices, and 1660–1700 cm^−1^ for β-turns [27]. Figure 4B shows that the relative content of quinoa protein secondary structure changed significantly after PEF treatment. Compared with N-QPI, the content of α-helix and β-turn in PEF-QPIs was significantly increased. After treatment with 30 pulses and 50 pulses at 7.5 kV/cm, the random coil of quinoa protein disappeared, and the content of β-sheets increased significantly. The results above indicated that PEF treatment (7.5 kV/cm 30 pulses) could transform the random coil structure of quinoa protein into a β-sheet structure and make the folding of quinoa protein more orderly, which might be related to the change in intermolecular and intramolecular forces [24]. Alavi et al.’s study showed that the percentage of β-sheets was related to the degree of protein aggregation [34]. However, high pulses (7.5 kV/cm 50 pulses) could reduce the ratio of α-helix and β-turn, resulting in less structural stability than that of the PEF-QPI 7.5 kV/cm 30 pulses. The results above indicated that the PEF treatment could change the secondary structure of quinoa proteins. The input energy of PEF treatment may increase the probability of protein molecules colliding, vibrating, and assembling, and improve the ordering and aggregation tendency of proteins. The conclusion above was consistent with the SEM scanning image analysis in this study.

### 3.5. Effects of PEF Treatment on the UV Spectrum, Intrinsic Fluorescence Spectrum, and Surface Hydrophobicity of QPIs

**Figure 5:**
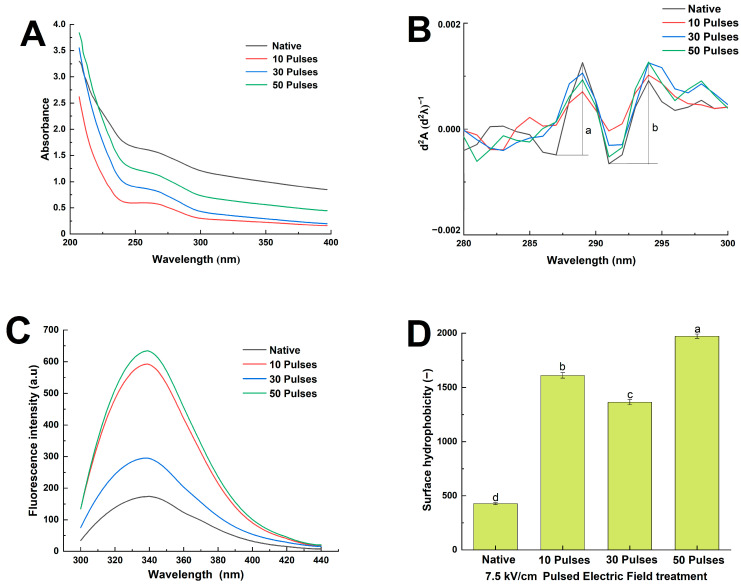
First-order UV spectrum (**A**), second-derivative UV spectrum (**B**), intrinsic fluorescence spectrum (**C**) (the peak value of 287–289 nm was “a”, and the valley value of 291–294 nm was “b”), and surface hydrophobicity (**D**) of N-QPI and 10, 30, and 50 pulses under 7.5 kV/cm treated PEF-QPIs, all experiments were repeated three times, and different letters indicate significant differences (*p* < 0.05).

The UV and intrinsic fluorescence spectra represented the tertiary structures of N-QPI and PEF-QPIs. The maximum absorption peaks of the UV spectra of tryptophan and tyrosine are usually at approximately 270 nm [28]. Figure 5A shows that N-QPI had an absorption peak at 257 nm, while PEF-QPIs had no significant redshift or blueshift, but had different UV spectral absorption intensities. By fitting the second derivative of the UV spectra, the peak-to-trough ratio (r = a/b) could be computed to speculate the effect of PEF treatment on the microenvironment of tyrosine and tryptophan residues and further deduce the change in the average polarity of the QPI [35]. According to Figure 5B, in the range of 280–300 nm, the index r of N-QPI was 1.109 and was significantly reduced to 0.596, 0.766, and 0.445 (7.5 kV/cm 10, 30, 50 pulses treatment), which certified that the average hydrophobicity significantly increased. Considering the average hydrophobicity of PEF-QPIs, that of the 7.5 kV/cm 30 pulses PEF-QPI was the lowest, but that of the 7.5 kV/cm 50 pulses PEF-QPI was the highest.

The intrinsic fluorescence spectrum is one of the main features of the microenvironment information of fluorescent chromogenic groups in protein molecules (tyrosine and tryptophan). A wavelength of 290 nm was chosen as the excitation wavelength to determine the effect of PEF treatment on the tertiary structure of quinoa proteins [28]. Figure 5C shows that the maximum emission wavelength (λ_max_) of N-QPI was 340 nm, while the maximum emission wavelength of PEF-QPIs was slightly blueshifted at 340 nm, indicating that PEF treatment changed the tertiary structure of quinoa protein, and the tryptophan residues transferred to the hydrophobic region increased the average hydrophobicity of quinoa protein [27]. The intrinsic fluorescence spectrum intensity of PEF-QPIs increased significantly from that of N-QPI (173.83 a.u), and the 7.5 kV/cm 50 pulses PEF treatment increased the highest to 634.17 a.u. The results indicated that the energy input of PEF treatment could expand the QPI molecule, thus exposing the buried hydrophobic groups in the protein core and increasing the peak fluorescence intensity. The decrease in fluorescence intensity after treatment with 7.5 kV/cm 30 pulses was relevant to the exposure and quenching of fluorophores inside the protein due to the alternating force generated by the polarity reversal of PEF treatment. As the pulses of PEF treatment increased, the energy input increased, and the peak increases indicated folding and rearrangement of the protein tertiary structure. The above pattern was similar to the intrinsic fluorescence spectra of quinoa proteins treated with sonication, studied by Zuo et al. [36]. The results above indicated that PEF treatment altered the tertiary structure of quinoa proteins, resulting in a change in the microenvironment of quinoa protein tryptophan and tyrosine, which was the same as the surface hydrophobicity change in this study. 

According to Figure 5D, the PEF treatment could significantly increase the surface hydrophobicity (H_0_) of quinoa protein up to 3.62 times compared with N-QPI. Cao’s study reported that the nonthermal treatment could shift a large amount of the hydrophobic groups inside the protein molecules to the surface and increase H_0_ [37]. The alternating force generated by polarity reversal in 7.5 kV/cm 10 pulses broke the complex structure of the protein, resulting in more hydrophobic regions exposed to the solvent environment. The QPI treated with 7.5 kV/cm 30 pulses partially aggregated under hydrophobic interactions. However, the 7.5 kV/cm 50 pulses treatment exposed more hydrophobic groups to quinoa proteins, causing a significant increase in H_0_. Zhang et al. also reported that the hydrophobicity of rice residue protein was increased significantly by nonthermal treatment such as high-pressure microfluidization [24]. The changes in the UV spectrum, the intrinsic fluorescence spectrum, and the surface hydrophobicity in this study indicated that PEF treatment could modify the tertiary structure of quinoa protein, which unfolded the structure of quinoa protein, exposed hydrophobic grouping and significantly increased H_0_.

### 3.6. Effects of PEF Treatment on the Thermal Stability of QPIs

**Figure 6:**
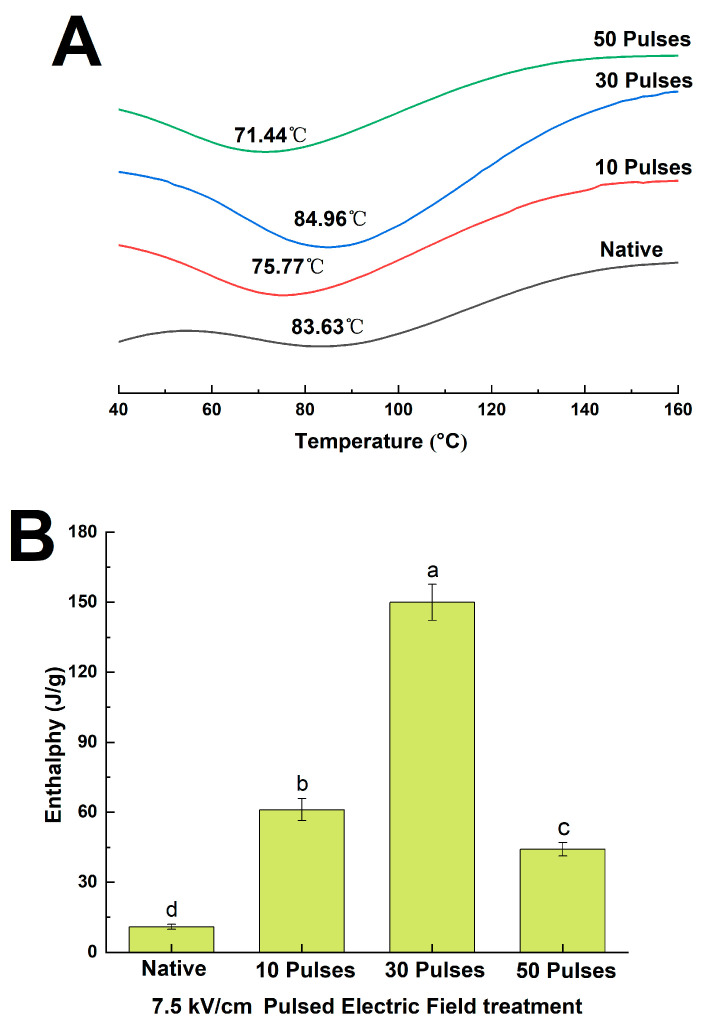
Differential scanning calorimetry (**A**), differential scanning calorimetry enthalpy value (**B**) of N-QPI and 10, 30, and 50 pulses under 7.5 kV/cm treated PEF-QPIs, all experiments were repeated three times, and different letters indicate significant differences (*p* < 0.05).

Figure 6A,B show the thermal stability of N-QPI and PEF-QPIs. T_d_ and ΔH usually represent the temperature and the quantity of heat needed for protein denaturation, respectively [24]. The T_d_ for N-QPI was 83.63 °C and the ΔH was 10.89 J/g. Similarly, Abugoch et al. [33] also observed a single endothermic peak in the DSC of QPI with T_d_ and ΔH values of only 83.4 °C and 0.96 J/g, respectively. The T_d_ of PEF-QPI in 7.5 kV/cm 30 pulses was 84.96 °C, 1.59% higher than that of N-QPI. T_d_ generally reflects the maintenance of the molecular structure of a protein, so higher T_d_ values in PEF treatment indicate increased structural compactness [38]. Compared with N-QPI, the ΔH of PEF-QPIs increased 12.77 times more (7.5 kV/cm 30 pulses), indicating that PEF treatment increased the heat needed for denaturation and significantly enhanced its thermal stability. However, the T_d_ and ΔH of QPI in the 7.5 kV/cm 50 pulses group decreased by 15.91% and 70.57%, respectively, compared to the 7.5 kV/cm 30 pulses group, destroying the thermal stability of QPI. The results above indicated that PEF treatment resulted in a more stable protein structure with higher thermal stability.

### 3.7. Effect of PEF Treatment on the Functional Properties of QPIs

#### 3.7.1. Effect of PEF Treatment on the Solubility of QPIs

**Figure 7:**
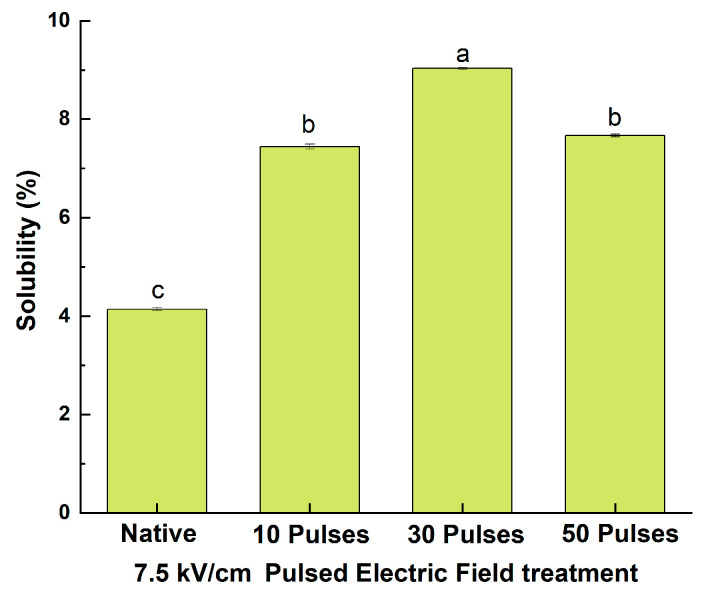
Solubility of N-QPI and 10, 30, and 50 pulses under 7.5 kV/cm-treated PEF-QPIs. All experiments were repeated three times, and different letters indicate significant differences (*p* < 0.05).

Solubility is one of the functional indicators of protein denaturation and valuable in potential commercial applications of food and beverage products [27]. According to Figure 7, the PEF treatment significantly increased the solubility of the QPIs. Compared with N-QPI, the solubility increased by 79.76%, 118.12%, and 85.24% with PEF treatment at 7.5 kV/cm of 10, 30, and 50 pulses, respectively. The results above may be due to PEF treatment destroying the noncovalent bond (such as the hydrogen bonding and the hydrophobic interaction forces) of quinoa protein particles, resulting in smaller size, increased absolute potential, inhibiting protein aggregation, and increased solubility [39]. However, the alternating force generated by 50 pulses caused some quinoa proteins to form aggregates, which reduced their solubility. In general, the more aggregates there are in a protein, the lower the solubility [40]. The results above showed that the PEF treatment could influence the solubility of quinoa protein by changing its particle size and intermolecular force.

#### 3.7.2. Effect of PEF Treatment on Foaming and Emulsifying Properties of QPIs

**Figure 8:**
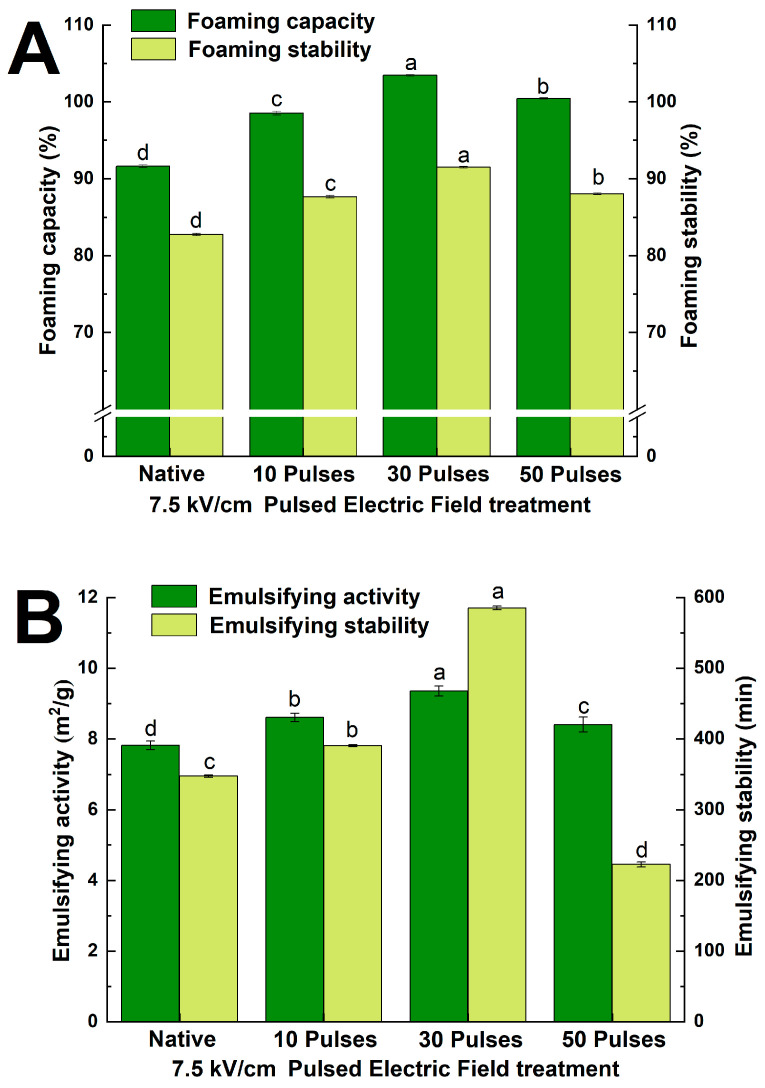
Foaming capacity and foaming stability (**A**), emulsifying activity index and emulsion stability index (**B**) of N-QPI and 10, 30, and 50 pulses 7.5 kV/cm-treated PEF-QPIs. All experiments were repeated three times, and different letters indicate significant differences (*p* < 0.05).

The foaming capacity of protein refers to the ability to stabilize bubbles by forming stiffer interfacial layers at the air–liquid interface. Foaming stability refers to the variation in foam volume per unit time [41]. According to Figure 8A, the foaming capacity and stability of the 7.5 kV/cm 30 pulses PEF treatment increased by 12.89% and 10.56%, respectively, compared with N-QPI. The foaming capacity depends on the solubility and surface hydrophobicity of the protein, which migrates the protein to the bubble surface and expands to form an interfacial layer at the air–liquid interface, resulting in a significant increase in protein foaming performance [25]. However, as the pulses increased (7.5 kV/cm 50 pulses), the alternating force generated by PEF treatment polarity reversal increased the interaction between protein particles and decreased the solubility of the protein, resulting in less protein adsorbed on the air-water interface and decreased foaming properties [41]. Similarly, PEF treatment exposed the hydrophobic groups, and the higher hydrophobic interaction promoted QPIs to form stiffer interfacial layers at the air-water interface than N-QPI. However, PEF treatment with excessive pulses (7.5 kV/cm 50 pulses) caused aggregation and reduced the foaming stability of QPI [42]. The results above indicated that the PEF treatment promoted the adsorption of quinoa proteins at the air-water interface and the formation of stiffer interfacial layers, which significantly improved the foaming capacity and foaming stability of QPI. 

EAI is the ability of the protein to adsorb at the oil-water interface during emulsion preparation, and ESI is the ability to keep the emulsion stable of the protein [28]. Figure 8B shows that the highest increase of PEF-QPIs emulsification was 19.63%, treated by 7.5 kV/cm 30 pulses. Zhang et al.’s study suggested that the improvement of emulsification was related to higher solubility and surface hydrophobicity [24]. PEF treatment reduced the particle size of QPI and accelerated the solubility and diffusion rate of QPI at the oil-water interface. PEF treatment also increased the surface hydrophobicity of QPI. The exposure of hydrophobic residues increases the interaction of QPI with water and decreases its adsorption force and tension at the oil-water interface. The PEF treatment elevated the balance of the hydrophilic and hydrophobic ratio and increased the EAI of QPI [43]. Liu et al. [28] observed a similar improvement in the EAI of *Zanthoxylum* seed protein modification by ultrasonication. Similarly, Zhao et al.’s study showed that excessive intensity of high-pressure homogenization treatment reduced the EAI of quinoa proteins [39]. The trends of ESI resembled those of EAI; PEF treatment increased the ESI of QPI remarkably, and after the 7.5 kV/cm 30 pulses treatment it was 68.41% higher than that of N-QPI. A possible explanation was that PEF treatment resulted in increased β-sheet content and improved the structural compactness of QPI, contributing to maintaining the structural integrity of the QPI during emulsion formation and storage, therefore improving the EAI of QPI [36]. Research by Zhang et al. on the characteristics of rice dreg protein isolate treated by high-pressure microfluidization showed it had a similar increase in ESI [24]. As the pulses increased (7.5 kV/cm 50 pulses), the alternating force increased the aggregation of QPI and decreased the solubility and the stability of the oil-water interfacial layers, and the ESI was 36.02% lower than that of N-QPI, which is analogous to the excessively treated QPI of high-pressure microfluidization [39]. The results above indicated that PEF treatment promoted the ability of QPI to adsorb at the oil-water interfacial layers and the stability to maintain emulsion, which significantly improved the EAI and ESI of QPI. 

## 4. Conclusions

The PEF treatment changed the structure of quinoa protein, reduced the particle size, increased the surface hydrophobicity, and improved the solubility of quinoa protein. In this process, the pulses of treatment were an essential factor. Fewer pulses (7.5 kV/cm 10, 30 pulses) induced the structure of QPI to expand and converted random coil into a β-sheet structure, which increased the stability and order of QPI. At the same time, the foaming capacity, foaming stability, emulsifying activity index, and emulsion stability index of QPI were significantly enhanced (*p* < 0.05). However, more pulses (7.5 kV/cm 50 pulses) resulted in restricted reaggregation of quinoa protein particles. Notably, PEF treatment at 7.5 kV/cm for 30 pulses resulted in a more stable structure and better functional properties of the QPI. In summary, PEF treatment enhanced the structural stability and functional properties of QPI, which made it a more promising nutritional plant protein and expanded the research value of PEF treatment in protein processing.

## Data Availability

The data that support the findings of this study are available on request from the corresponding author, X.W., upon reasonable request. The data are not publicly available due to laboratory policies and confidentiality agreements. We have fully described the experimental design, analysis, and results, as well as the process of data analysis and processing. If editors and reviewers have questions about specific data, we do our best to provide more detailed explanations and illustrations.
